# Supplementary material for: The Evolution and Characterization of the RNA Interference Pathways in Lophotrochozoa
Source: Genome Biol Evol. 2024 May 7;16(5):evae098. doi: 10.1093/gbe/evae098 (PMC11114477; doi:10.1093/gbe/evae098)
Supplement: evae098_Supplementary_Data [file evae098_supplementary_data.zip › Supplementary_materials.zip!/selected_miRNAs/NoGe_selected_miRNAs/mirdeep_pdf_files/NMRB01005527.1_Notospermus_geniculatus_strain_Ushimado_scaffold5527,_whole_genome_shotgun_sequence_47693.pdf]

Provisional ID	:	NMRB01005527.1_Notospermus_geniculatus_strain_Ushimado_scaffold5527,_whole_genome_shotgun_sequence_47693
Score total	:	119.1
Score for star read(s)	:	3.9
Score for read counts	:	113.3
Score for mfe	:	2.5
Score for randfold	:	0
Score for cons. seed	:	-0.6
Total read count	:	234
Mature read count	:	156
Loop read count	:	0
Star read count	:	78

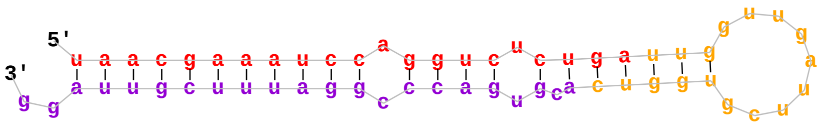

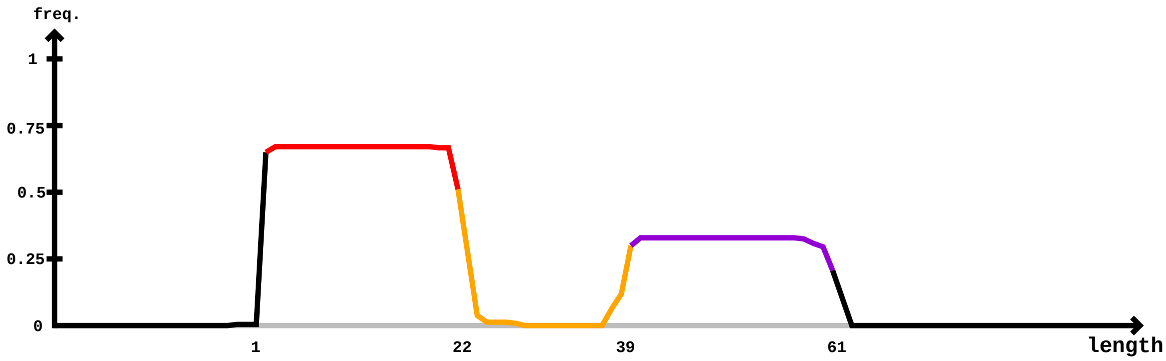


```
                                Mature                                Star
5'- gcgaugaccuguaccugucc uaacgaaauccaggucucuga uugguugauucgugguc acgugacccggauuucguuagg ccagguacagguacgucgacaagauggcggc  -3'   obs
```

```
5'- gcgaugaccuguaccuguccuaacgaaauccaggucucugauugguugauucguggucacgugacccggauuucguuaggccagguacagguacgucgacaagauggcggc   -3'   obs
    gcgaugaccuguaccuguccuaacgaaauccaggucucugauugguugauucguggucacgugacccggauuucguuaggccagguacagguacgucgacaagauggcggc         exp
    .((((((((((((((((.(((((((((((((.((((.(((((((..........)))))).).)))).))))))))))))).))))))))))).))))).............    reads   mm   sample
    .................uccuaacgaaaGccaggucucugauuggu.................................................................    1       1    Mm3
    ....................uaacgaaauccGggucucug.......................................................................    1       1    Mm3
    ....................uaacgaaauccaggucucug.......................................................................    24      0    Mm3
    ....................uaacgaaaucAaggucucuga......................................................................    1       1    Mm3
    ....................uaacgaaauccaggucucuga......................................................................    36      0    Mm3
    ....................uaacgaaauccaggucucugG......................................................................    2       1    Mm3
    ....................uaacgaaauccGggucucuga......................................................................    22      1    Mm3
    ....................uaacgaaGuccaggucucuga......................................................................    1       1    Mm3
    ....................uaacgaaauccaggucucugaA.....................................................................    4       1    Mm3
    ....................uaacgaaauccGggucucugau.....................................................................    4       1    Mm3
    ....................uaacgaaauccaggucCcugau.....................................................................    1       1    Mm3
    ....................uaacgaaauccaggucucugau.....................................................................    13      0    Mm3
    ....................uaacgaaGuccaggucucugau.....................................................................    1       1    Mm3
    ....................uaacgaaauccaggucAcugau.....................................................................    1       1    Mm3
    ....................uaacgaaauccaggucuUugau.....................................................................    1       1    Mm3
    ....................uaacgaaauccaggucucugaAu....................................................................    2       1    Mm3
    .....................aacgaaauccaggucucugauu....................................................................    1       0    Mm3
    ........................................................ucacgugacccggauuucguuag................................    2       0    Mm3
    ........................................................ucacgugacccggauuucguuagU...............................    1       1    Mm3
    .........................................................cacgugacccggauuucguuag................................    4       0    Mm3
    .........................................................cacgugacccggauuucAuuag................................    1       1    Mm3
    .........................................................cacgugacccggauuucguuagU...............................    2       1    Mm3
    ..........................................................acgugacccggauuucguuag................................    5       0    Mm3
    ..........................................................acgugacccggauuucguuagU...............................    5       1    Mm3
    ..........................................................acgugacccggauuucguuagg...............................    4       0    Mm3
    ..........................................................acgugacccggauuucgAuagg...............................    1       1    Mm3
    ..........................................................acgugacccggauuucguuagA...............................    3       1    Mm3
    ..........................................................acgugacccggauuucguuaggc..............................    7       0    Mm3
    ..........................................................acgugacccggauuucguuGggc..............................    3       1    Mm3
    ...........................................................cgugacccggauuucguuaggc..............................    4       0    Mm3

    ....................uGacgaaauccaggucucugau.....................................................................    5       1    Mm1
    ....................uaacgaaauccaggucucugau.....................................................................    2       0    Mm1
```

Mature **Star**

```
gcgaugaccuguaccuguccuaacgaaauccaggucucugauugguugauucguggucacgugacccggauuucguuaggccagguacagguacgucgacaagauggcggc

....................uaacgaaauccaggucuc.........................................................................   1    0    Mm2
....................uaacgaaauccaggucucug.......................................................................   12   0    Mm2
....................uaacgaaauccaggucucuga......................................................................   5    0    Mm2
....................uaacgaaauccGggucucuga......................................................................   5    1    Mm2
....................uaacgaaGuccaggucucuga......................................................................   2    1    Mm2
....................uaacgaaauccGggucucugau.....................................................................   1    1    Mm2
....................uaacgaaauccaggucucugaA.....................................................................   2    1    Mm2
....................uaacgaaauccaggucucugau.....................................................................   1    0    Mm2
....................uaacgaaauccGggucucugauu....................................................................   1    1    Mm2
....................uaacgaaauccGggucucugauugguu................................................................   1    1    Mm2
....................uaacgaaaGccaggucucugauugguu................................................................   1    1    Mm2
.....................aacgaaauccGggucucugau.....................................................................   1    1    Mm2
.....................aacgaaauccaggucucugauu....................................................................   2    0    Mm2
........................................................ucacgugacccggauuucguua.................................   2    0    Mm2
........................................................ucacgugacccggauuucguuU.................................   1    1    Mm2
........................................................ucacgCgacccggauuucguuag................................   1    1    Mm2
........................................................ucacgugacccggauuucguAag................................   1    1    Mm2
.........................................................cacgugacccggauuucgu...................................   1    0    Mm2
.........................................................cacgCgacccggauuucguuag................................   1    1    Mm2
.........................................................cacgugacccggauuuUguuagg...............................   1    1    Mm2
.........................................................cacgugacccggauuucguuagA...............................   1    1    Mm2
..........................................................acgugacccggauuucguuag................................   1    0    Mm2
..........................................................acgCgacccggauuucguuag................................   2    1    Mm2
..........................................................acgCgacccggauuucguuagg...............................   6    1    Mm2
..........................................................acgugacccggauuucguuagg...............................   5    0    Mm2
..........................................................acgugacccggauuucguuaggc..............................   1    0    Mm2
...........................................................cgugacccggauuucguuaggc..............................   3    0    Mm2

.....................aacgaaauccaggucucuga......................................................................   1    0    Fm3
........................................................ucacgugacccggauuucguu..................................   4    0    Fm3
........................................................ucacgugacccggauuucguuaU................................   3    1    Fm3
.........................................................cacgugacccggauuucguuagU...............................   2    1    Fm3
```
